# Prehospital stroke notification and endovascular therapy for large vessel occlusion: a retrospective cohort study

**DOI:** 10.1038/s41598-022-14399-0

**Published:** 2022-06-16

**Authors:** Satoru Fujiwara, Takehito Kuroda, Yoshinori Matsuoka, Nobuyuki Ohara, Hirotoshi Imamura, Yosuke Yamamoto, Koichi Ariyoshi, Nobuo Kohara, Michi Kawamoto, Nobuyuki Sakai

**Affiliations:** 1 Department of Neurology, Kobe City Medical Center General Hospital, Kobe, Japan; 2 Department of Cerebrovascular Medicine and Neurology, National Cerebral and Cardiovascular Center, Suita, Japan; 3 Department of Emergency Medicine, Kobe City Medical Center General Hospital, 2-1-1 Minatojima-Minamimachi, Chuo-ku, Kobe, Hyogo 650-0047 Japan; 4 Department of Healthcare Epidemiology, Graduate School of Medicine and Public Health, Kyoto University, Kyoto, Japan; 5 Department of Neurosurgery, Kobe City Medical Center General Hospital, Kobe, Japan

**Keywords:** Medical research, Stroke

## Abstract

The impact of prehospital notification by emergency medical services (EMS) on outcomes of endovascular therapy (EVT) for large vessel occlusion (LVO) remains unclear. We therefore explored the association between prehospital notification and clinical outcomes after EVT. In this single-center retrospective study from 2016 through 2020, we identified all LVO patients who received EVT. Based on the EMS’s usage of a prehospital stroke notification system, we categorized patients into two groups, Hotline and Non-hotline. The primary outcome was good neurological outcome at 90 days; other time metrics were also evaluated. Of all 312 LVO patients, the proportion of good neurological outcomes was 94/218 (43.1%) in the Hotline group and 8/34 (23.5%) in the Non-hotline group (adjusted odds ratio 2.86; 95% confidence interval 1.12 to 7.33). Time from hospital arrival to both tissue plasminogen activator and to groin puncture were shorter in the Hotline group (30 (24 to 38) min vs 48(37 to 65) min, p < 0.001; 40 (32 to 54) min vs 76 (50 to 97) min, p < 0.001), respectively. In conclusion, prehospital notification was associated with a reduction in time from hospital arrival to intervention and improved clinical outcomes in LVO patients treated with EVT.

## Introduction

In the era of endovascular therapy (EVT) and tissue plasminogen activator (t-PA) for stroke patients with large vessel occlusion (LVO), the time from onset to treatment has become more critical than ever. These interventions are essentially time-sensitive^1–4^, and so every health care provider should pay great attention to this factor. The chain of care for stroke begins in the prehospital setting^5^, and therefore to shorten the time taken, the role of emergency medical services (EMS) has been increasing recently. EMS providers need to suspect the possibility of stroke appropriately at the scene and transport patients as quickly as possible to appropriate hospitals, such as those with EVT-capable facilities. Furthermore, prehospital notification by EMS is recommended by the American Heart Association guidelines, as it is considered to shorten the time after arrival at hospital to treatment^6^. Thus, seamless treatment from the prehospital to the in-hospital setting is key to improving clinical outcomes in stroke patients with LVO.

Several studies have already reported the usefulness of prehospital notification by EMS to receiving hospitals both in reducing the time from hospital arrival to t-PA therapy, and also in improving rates of administration of t-PA^7–9^. These goals are considered achievable, as prenotification by EMS allows medical staff to prepare imaging devices such as computed tomography, or activate the hospital’s stroke team before the patient’s arrival^10^. Although these recent studies tried to evaluate the effect of prehospital notification on patient’s clinical outcomes, they did not succeed in actually proving the point. Further, there has been little evidence regarding stroke patients with LVO who are treated with EVT, not with t-PA alone. Additionally, the characteristics of LVO patients, without the EMS having suspected stroke, must be helpful in refining prehospital stoke management, but there has been insufficient data on the clinical characteristics, frequency and outcomes of these patients.

The present study aimed to examine the association between prehospital notification by EMS and both clinical outcomes of LVO patients and time metrics, and, additionally, to describe the characteristics of LVO patients transported without suspicion of stroke.

## Methods

### Study design and setting

We conducted a single-center retrospective study at Kobe City Medical Center General Hospital, Kobe, Japan from May 2016 through March 2020. Kobe City Medical Center General Hospital is a tertiary referral hospital with a 768-bed capacity. It has an emergency department receiving an average of 35,000 patient visits and 10,000 ambulance arrivals per year. As the city has a population of 1.5 million inhabitants living in a relatively small area of 557 km^2^, where EMS can transport any patient directly to our hospital within an hour, interhospital transfers between stroke centers, using, for example, the drip and ship strategy, are rare.

The study adhered to the Declaration of Helsinki guidelines for medical research involving human subjects. The local Institutional Review Board of Kobe City Medical Center General Hospital approved the study protocol (k200304) and permitted to waive written informed consent, as this retrospective study used clinical information obtained from daily practice, which does not contain patient-identifiable data.

### Prehospital stroke management and acute stroke protocol in our hospital

In 2002, we launched a ‘no-refusal stroke hotline’ which enables the EMS to contact a stroke physician directly on the phone, thereby reducing the on-scene time required for selecting hospitals to transport patients to. Thus, for twenty years, we have been accepting almost all patients who have been suspected of stroke under the policy of “anyone, anytime”. To refine the prehospital stroke system, we have a monthly review with the Kobe City Fire Bureau, as in Japan, it is the local city fire department that provides the initial EMS. We discuss all cases and provide feedback to the EMS, especially regarding their diagnosis at the scene and the final diagnosis after arriving at the hospital.

**Figure 1:**
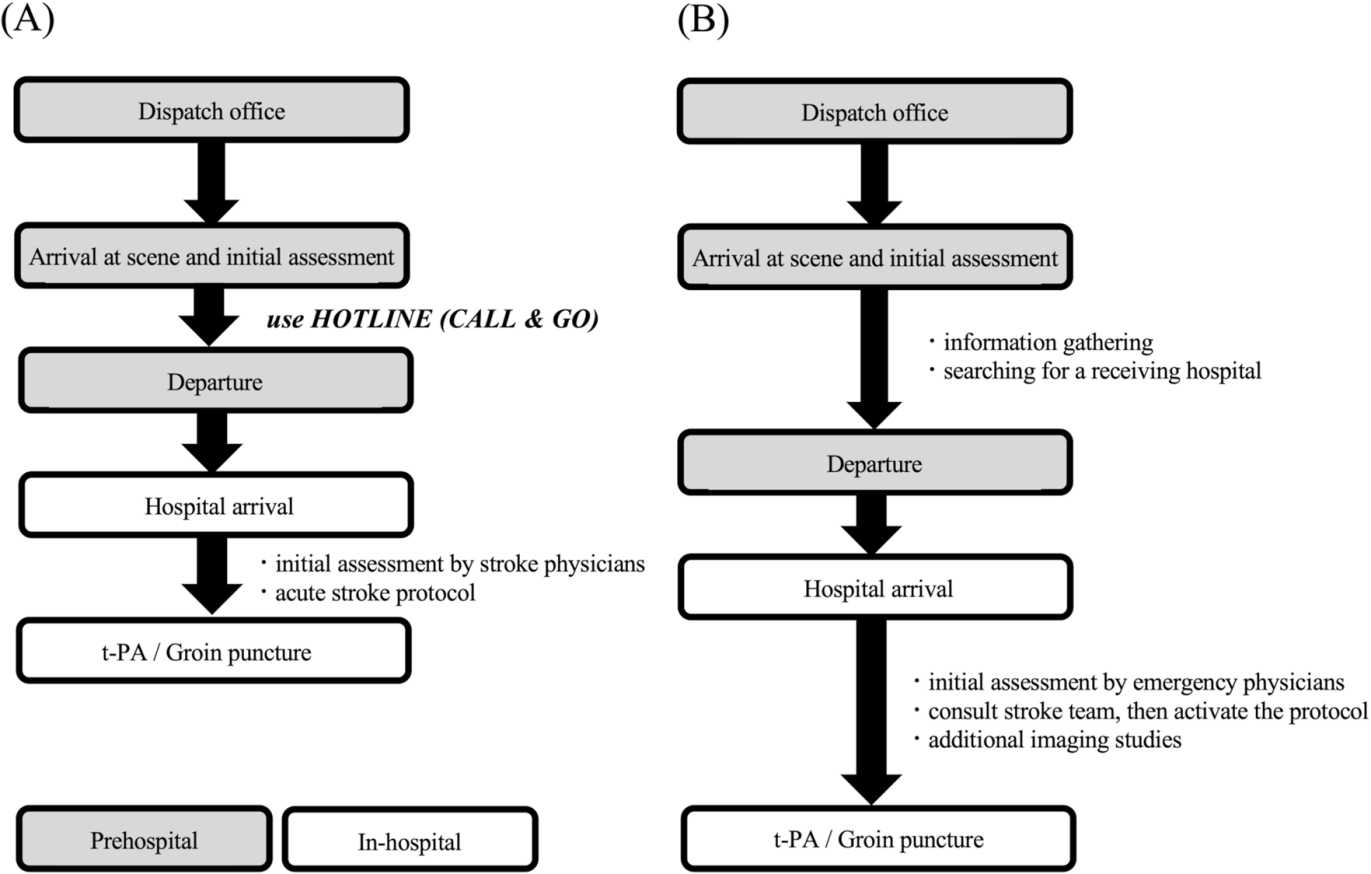
Time courses in management of large vessel occlusion patients with or without using a stroke hotline. (**A**) When emergency medical services (EMS) suspected a patient of having a stroke, the stroke hotline allows the EMS to depart the scene as soon as possible, and they do not need to spend their time searching for a receiving hospital. Medical staff in the hospital are also able to prepare tissue plasminogen activator (t-PA) and endovascular therapy (EVT) based on the prehospital information. Furthermore, we have developed a stroke protocol to shorten the time from the patient’s arrival to stroke treatments. (**B**) When a patient is not suspected of having a stroke, the EMS need to spend their time at the scene searching for a receiving hospital, leading to a longer prehospital time. Further, after arriving at the hospital, the stroke protocol is not activated until emergency physicians complete their initial assessment of the patient and consult a stroke physician. Consequently, it often takes longer from arrival at the emergency department to undergoing initial stroke care.

The flowcharts demonstrate pre- and in-hospital stroke management according to whether the EMS utilized the stroke hotline (Fig. 1A) or not (Fig. 1B). When EMS suspected a patient of having any stroke during their initial evaluation, they can depart the scene quickly using the stroke hotline. For these patients, we can predict an indication for t-PA and EVT based on the information provided, and then activate the stroke protocol before the patient’s arrival, which enables us to shorten the in-hospital time to treatment (Fig. 1A). The key features of the stroke protocol in our hospital are as follows:

1. We use discrete online services and the hospital's communication network to convene the medical staff necessary for initial care and treatment: at least two stroke physicians are available for patient arrivals at any given time.
2. We use rapid measurement kits for PT-INR, immediately after the patient's arrival so as to start treatment without delay.

On the other hand, when patients are not suspected of having stroke, the EMS need to search for a receiving hospital to transport the patient to and ask them to accept the patient, which, in most cases, takes a longer time than using the stroke hotline, especially when they need to contact multiple hospitals. Also, after arriving at our hospital, emergency physicians first provide an initial evaluation and medical care. Only after they believe that the patient has had a stroke can they call for a stroke physician and we can then activate the protocol. As a result, the initiation of t-PA or EVT is often delayed (Fig. 1B).

### Selection of participants

We analyzed all LVO patients who were transported to our hospital and underwent EVT. We excluded patients for whom we had insufficient information—for example, patients whose emergency triage sheets, or data regarding outcomes and time metrics could not be found in electronic medical records. We also excluded patients lost to follow up, patients who were not involved in an initial assessment by the EMS, such as cases transferred from nearby medical institutions or cases of exacerbation or recurrence after hospitalization.

### Data collection

We reviewed the electronic medical records and EMS run-sheets of eligible patients. The following data were collected: patient characteristics (age, sex, pre-stroke modified Rankin Scale (mRS)^11^), past medical history (hypertension, diabetes mellitus, dyslipidemia, coronary artery disease, chronic renal failure), findings on admission (systolic blood pressure, National Institute of Health Stroke Scale (NIHSS)^12^, Alberta Stroke Program Early CT Score (ASPECTS), occluded vessel, and stroke classification)^13^, rates of administration of t-PA, clinical outcomes at 90 days after onset, time metrics in the management of LVO (the time from hospital arrival to t-PA, from hospital arrival to groin puncture, and from hospital arrival to recanalization), the rates of successful recanalization (modified thrombolysis in cerebral infarction (TICI) grade^14^ ≥ 2b), and mortality at 90 days. ASPECTS in this study was assessed by preoperative CT in the case of anterior circulation and by diffusion-weighted imaging on MRI in the case of posterior circulation^15^. Recanalization time was defined as the time by which modified TICI grade ≥ 2b was achieved.

### Hotline vs. non-hotline

We divided the participants into Hotline and Non-hotline groups. In the Hotline group, the EMS suspected the patients of having had a stroke and transported them using the stroke hotline. On the other hand, in the Non-hotline group, patients were not suspected of having had a stroke and the stroke hotline was not activated.

### Outcome measures

The primary outcome was good neurological outcome at 90 days after onset. Good neurological outcome was defined as patients with an mRS of 0–2 at 90 days after onset of symptoms, or patients whose mRS at 90 days after onset of symptoms was the same as the mRS before admission if the mRS before onset was 3 or higher^16^.

The secondary outcomes were time metrics in the management of LVO (time from hospital arrival to t-PA, hospital arrival to groin puncture, and hospital arrival to recanalization), the rates of administration of t-PA, and successful recanalization (modified TICI grade ≥ 2b).

In addition to these outcomes, we also outlined details of clinical features in the Non-hotline group, and assessed the reasons why the EMS did not suspect stroke at the scene.

### Statistical analysis

We presented the number (percentage) for categorical variables, and medians (interquartile ranges (IQRs)) for continuous variables. We compared patient characteristics as well as clinical outcomes between the Hotline and Non-hotline groups using χ^2^ test for categorical variables and Mann–Whitney U test for continuous variables.

In the primary analysis, we constructed a logistic regression model using a complete data set. We compared primary outcomes between the two groups, adjusting for variables as follows: age (< 65 years, 65 to 74, ≥ 75 years), sex, pre-stroke mRS, NIHSS, ICA and M1occlusion, or VA and BA occlusion. We classified age groups as ≤ 64 years, ≤ 65 years to < 75 years (early elderly), and ≥ 75 years (late elderly) based on the criteria for advanced age used by the Japanese health insurance system. Other variables for the logistic model were selected based on biological plausibility and preexisting knowledge.

All statistical analyses were performed by using STATA version 15.1 (StataCorp, College Station, TX). In all hypothesis tests, values of *P* < 0.05 were considered statistically significant.

## Results

### Participants and baseline characteristics

**Figure 2:**
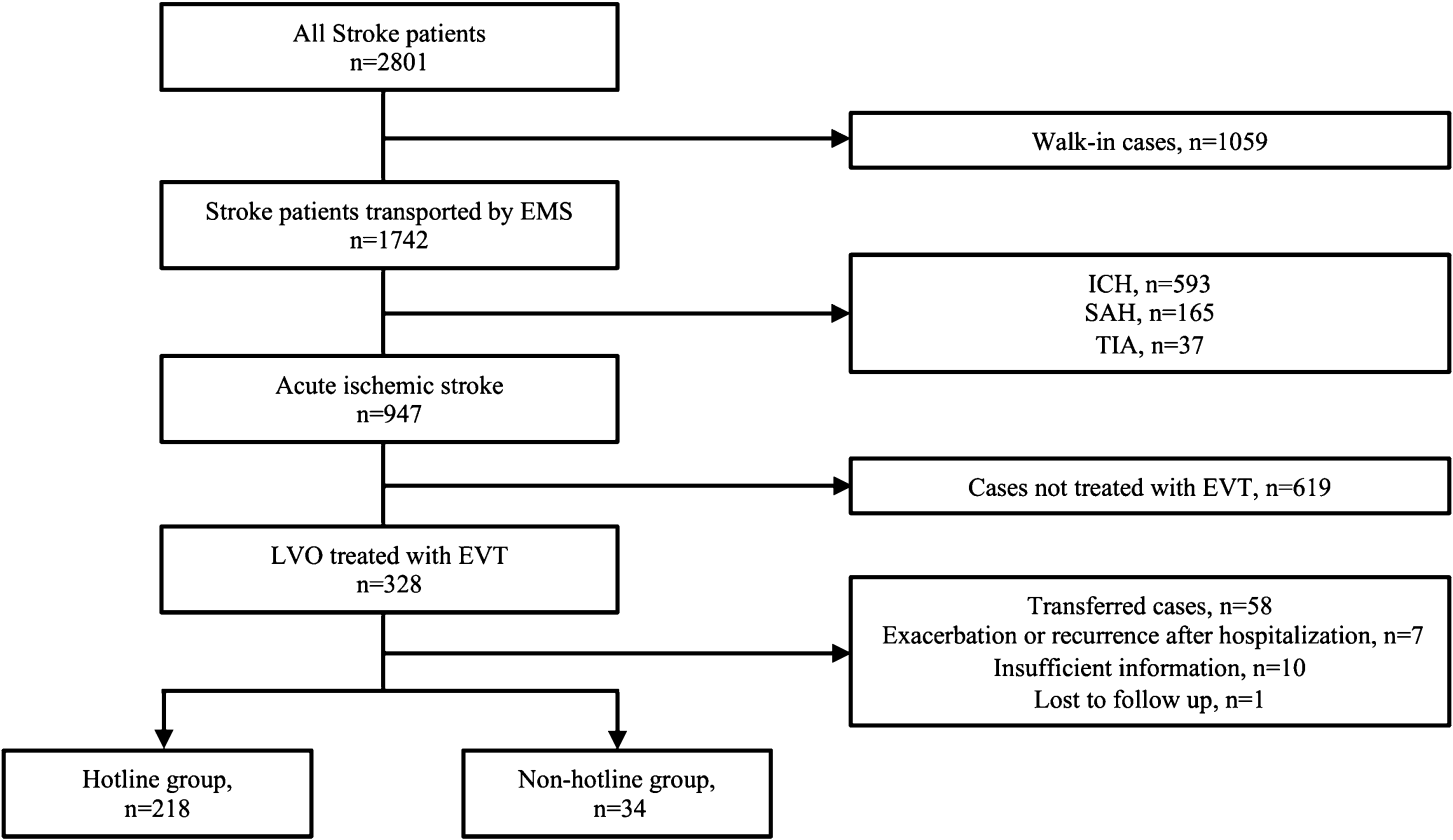
Study flowchart. *EMS* emergency medical services, *EVT* endovascular therapy, *ICH* intracranial hemorrhage, *SAH* subarachnoid hemorrhage, *TIA* transient ischemic attack.

A total of 2801 stroke patients were hospitalized from March 2016 through May 2020, and 328 patients were treated with EVT for LVO. After excluding ten patients with insufficient information, 58 patients transferred from other hospitals, seven patients with exacerbation or recurrence after hospitalization, and one patient lost to follow up, we included 252 patients in our analyses. Of these patients, 218 patients (86.5%) were transported using a hotline (Hotline group), and 34 patients (13.5%) were transported without the use of a hotline (Non-hotline group) (Fig. 2).

Table 1 shows the baseline characteristics of stroke patients in the Hotline and Non-hotline group (Table 1).

**Table 1:** Clinical characteristics of patients with large vessel occlusion who received endovascular therapy: hotline group vs. non-hotline group.

	Total (n = 252)	Hotline group (n = 218)	Non-hotline group (n = 34)	P value
**Age, median (IQR), years**	79 (70 to 85)	78 (70 to 85)	81 (72 to 85)	0.61
≤ 64 years, n (%)	37 (14.7)	33 (15.1)	4 (11.8)	
65 to 74 years, n (%)	63 (25.0)	55 (25.2)	8 (23.5)	
≥ 75 years, n (%)	152 (60.3)	130 (59.6)	22 (64.7)	
Male, n (%)	142 (56.3)	122 (56.0)	20 (58.8)	0.75
mRS before onset, median (IQR)	0 (0 to 2.5)	0 (0 to 2)	0 (0 to 3)	0.68
**Past medical history**				
Hypertension^a^, n (%)	134 (53.4)	121 (55.8)	13 (38.2)	0.06
Diabetes mellitus^a^, n (%)	44 (17.5)	41 (18.9)	3 (8.8)	0.15
Dyslipidemia^a^, n (%)	53 (21.1)	48 (22.1)	5 (14.7)	0.33
Coronary artery disease^a^, n (%)	29 (11.6)	25 (11.5)	4 (11.8)	0.97
Chronic renal failure, n (%)	25 (9.9)	23 (10.6)	2 (5.9)	0.4
Symptom onset or last known well to hospital arrival, median (IQR), min	87 (50 to 269)	83 (46 to 257)	148 (60 to 355)	0.30
Systolic blood pressure^b^, median (IQR), mmHg	155 (136 to 173)	156 (140 to 174)	146 (130 to 166)	0.04
NIHSS on admission, median (IQR)	19 (13 to 26)	19 (14 to 26)	19 (12 to 28)	0.93
ASPECTS on admission^c^, median (IQR)	9 (7 to 10)	10 (7 to 10)	8 (8 to 10)	0.18
**Occluded vessel**				
ICA & M1, n (%)	169 (67.1)	152 (69.7)	17 (50.0)	0.023
Posterior circulation, n (%)	24 (9.5)	16 (7.3)	8 (23.5)	0.003
Anterior circulation, right, n (%)	120 (47.6)	106 (48.6)	14 (41.2)	0.42
Anterior circulation, left, n (%)	110 (43.7)	97 (44.5)	13 (38.2)	0.49
**Stroke classification**				0.63
Cardioembolic, n (%)	151 (60)	128 (58.7)	23 (67.6)	
Atherothrombotic, n (%)	44 (17.5)	39 (17.9)	5 (14.7)	
Stroke of undetermined etiology, n (%)	50 (19.8)	44 (20.2)	6 (17.6)	
Other, n (%)	7 (2.8)	7 (3.2)	0 (0)	

*IQR* interquartile range, *mRS* modified Rankin Scale, *NIHSS* National Institutes of Health Stroke Scale, *ASPECTS* Alberta stroke program early computed tomography score, *ICA* internal carotid artery, *M1* M1 segment of middle cerebral artery.

Data were available for 251 patients^a^, 248 patients^b^, and 237 patients^c^.

The median age for all patients was 79 (IQR, 70 to 85) years, and 56.3% of the patients were male. There was no clinically meaningful difference in age, sex, mRS before onset, systolic blood pressure, NIHSS, and ASPECTS and stroke classification. In the Hotline group, with the exception of coronary artery disease, more patients had comorbidities, such as hypertension, diabetes mellitus, dyslipidemia, and chronic renal failure. There were more ICA and M1 occlusions and fewer VA-BA occlusions in the Hotline group than in the Non-hotline group. Furthermore, patients in the Hotline group were less likely to have cardioembolism and stroke of undetermined etiology and more atherothrombotic stroke compared with those in the Non-hotline group.

### Primary outcomes

Good neurological outcomes at 90 days were significantly higher in the Hotline group than in the Non-hotline group (43.1% vs 23.5%, p = 0.030) (Table 2).

**Table 2:** Clinical outcomes in patients with large vessel occlusion who received endovascular therapy: Hotline group vs. Non-hotline group.

	Total (n = 252)	Hotline group (n = 218)	Non-hotline group (n = 34)	P value
**Primary outcome**				
Good neurological outcome at 90 days, n (%)	102 (40.5)	94 (43.1)	8 (23.5)	0.030
**Secondary outcomes**				
Hospital arrival to t-PA time^a^, median (IQR), min	31 (24 to 41)	30 (24 to 38)	48 (37 to 65)	< 0.001
Hospital arrival to groin puncture time, median (IQR), min	42 (33 to 57)	40 (32 to 54)	76 (50 to 97)	< 0.001
Hospital arrival to recanalization time, median (IQR), min	90 (69 to 136)	88 (67 to 127)	121 (83 to 176)	0.003
t-PA use, n (%)	141 (56.0)	127 (58.3)	14 (41.2)	0.062
Successful recanalization (modified TICI2b-3), n (%)	225 (89.3)	193 (88.5)	32 (94.1)	0.33

*IQR* interquartile range, *T-PA* tissue plasminogen activator, *TICI* thrombolysis in cerebral infarction, *ICH* intracranial hemorrhage.

^a^Only stroke patients who were treated with t-PA were included.

In multivariate analysis, hotline usage for prehospital notification was associated with good neurological outcome at 90 days (adjusted odds ratio (OR): 2.86, 95% CI: 1.12 to 7.33) (Table 3). Compared to patients under 65 years of age, older age showed a less-favorable neurological outcome at 90 days, with an adjusted OR of 0.56 (95% CI: 0.22 to 1.47) in early older patients and 0.18 (95% CI: 0.07 to 0.45) in late elderly patients. Likewise, a higher NIHSS was associated with a less-favorable neurological outcome at 90 days, with an adjusted OR of 0.93 (95% CI: 0.90 to 0.97).

**Table 3:** Multivariate logistic regression analysis for good neurological outcome in patients with large vessel occlusion who received endovascular therapy.

Variables	Odds ratio	95% confidence interval
Hotline-group	2.86	1.12 to 7.33
**Age (category)**		
< 65	Reference	
65 to 74	0.56	0.22 to 1.47
≥ 75	0.18	0.07 to 0.45
Sex	1.54	0.81 to 2.92
mRS before onset	0.81	0.63 to 1.04
NIHSS on admission	0.93	0.90 to 0.97
ICA and M1 occlusion	0.68	0.36 to 1.29

We calculated adjusted odds ratios for good neurological outcomes using a multivariate logistic model, in which we selected variables as follows: age (< 64, 65 to 74, ≥ 75 years), sex, mRS before onset, NIHSS in admission, ICA and M1 occlusion.

*mRS* modified Rankin Scale, *NIHSS* National Institutes of Health Stroke Scale, *ICA* internal carotid artery, *M1* M1 segment of middle cerebral artery.

### Secondary and Safety outcomes

Table 2 shows secondary and safety outcomes for stroke patients with large vessel occlusion who received EVT.

All time metrics were significantly shorter in the Hotline group than the Non-hotline group: hospital arrival to t-PA time (30 (24 to 38) min vs 48 (37 to 65) min; p < 0.001); hospital arrival to groin puncture time (40 (32 to 54) min vs 76 (50 to 97) min; p < 0.001); and hospital arrival to recanalization time (88 (67 to 127) min vs 121 (83 to 176) min; p = 0.003). Stroke patients in the Hotline group were more likely to have administration of t-PA than those in the Non-hotline group (58.3% vs 41.2%), though this was not statistically significant.

### Clinical features of patients in the Non-hotline group

Table 4 shows details of clinical features in the Non-hotline group, and reasons why the EMS could not use the stroke hotline. Among them, conjugate deviation was overlooked in 15 patients (44.1%), aphasia in ten patients (29.4%), and unilateral spatial neglect in 8 patients (23.5%). The EMS transported patients without using the stroke hotline because they prioritized differential diagnoses other than stroke despite recognizing neurological findings in 16 patients (47.1%); or they simply overlooked typical cortical symptoms indicative of LVO in nine patients (26.5%); or they missed nine other patients (26.5%) with either convulsive movements or who were comatose and lacked any neurological findings.

**Table 4:** Clinical characteristics of patients with large vessel occlusion who received endovascular therapy transported to the emergency department without the use of a stroke hotline.

**Characteristics of the non-hotline group (N = 34)**	
Age, median (IQR), years	81 (72 to 85)
Men, n (%)	20 (58.8)
mRS before onset 0–1, n (%)	22 (64.7)
GCS evaluated on scene^a^, median (IQR)	11 (6 to 14)
NIHSS on admission, median (IQR)	19 (12 to 28)
Proportion of missed neurological deficits by EMS, n (%)	26 (76.5)
**Details of neurological deficits missed by EMS on scene**	
Conjugate deviation, n (%)	15 (44.1)
Aphasia, n (%)	10 (29.4)
Unilateral spatial neglect, n (%)	8 (23.5)
Extinction, n (%)	4 (11.8)
Sensory disturbance, n (%)	4 (11.8)
Apraxia, n (%)	2 (5.9)
Other^b^, n (%)	7 (20.6)
**Main reasons why EMS transported patients without using the stroke hotline**	
Other differential diagnosis prioritized despite neurological deficit, n (%)	16 (47.1)
Failure to recognize cortical symptoms, n (%)	9 (26.5)
Epileptic seizure or comatose status without suspecting stroke, n (%)	9 (26.5)

*IQR* interquartile range, *mRS* modified Rankin Scale, *GCS* Glasgow Coma Scale, *NIHSS* National Institutes of Health Stroke Scale, *EMS* emergency medical services.

^a^Data were available for 31 patients.

^b^Other neurological deficits included hemiplegia (5 cases), decerebrate posturing (1 case), and convulsion (1 case).

## Discussion

In the analysis of our single center retrospective cohort, we demonstrated that proper prehospital notification of stroke by the EMS was associated with better neurological outcome at 90 days after onset, as well as shorter time metrics, such as hospital arrival to t-PA time and hospital arrival to groin puncture time. Additionally, we outlined the clinical features of LVO patients who were transported without the supposition of stroke and discussed the reasons why the EMS failed to consider the possibility of stroke at the scene.

The present study demonstrated the possibility that prehospital notification not only improved time metrics related to stroke management, but also led to better patient outcomes. Previous studies already showed that prehospital notification reduced time metrics associated with t-PA administration in stroke patients^7–9^. Our study confirmed the additional merit of prehospital notification, namely, that it improved both time to t-PA administration and time to groin puncture. These effects are reasonably explained by the idea that prehospital notification by the EMS can activate stroke protocols and the catheter laboratory, and allow the stroke team to prepare for the patient’s arrival. Furthermore, in contrast to previous studies, prenotification by the EMS was favorably associated with patients’ actual clinical outcomes. The main difference between these studies might be whether patients received EVT or not, though there are also several disparities such as the study design and the countries where the studies were conducted. We believe that in the era of t-PA alone prehospital notification was insufficient to alter patient outcomes, but the advent of EVT has changed the situation. Consequently, the importance of the EMS’s role and prehospital notification seems to be increasing as stroke management has become more and more effective and time-sensitive.

The clinical features of LVO patients whom the EMS failed to recognize as having had a stroke at the scene can play a critical role in refining the usage of stroke hotlines and prehospital stroke management but, unfortunately, such features have not been well documented in the literature. The usefulness of several prehospital scales in identifying LVO has been established^17–20^, but only those patients for whom the EMS successfully considered the possibility of stroke have the chance to benefit from such scales. Therefore, to add further knowledge to this issue, we assessed and discussed the three reasons why the EMS team transported stroke patients without using a hotline in this study. First, they were unable to prioritize stroke while considering other differential diagnoses such as head trauma and aortic dissection. Under this scenario, we have to emphasize the importance of prehospital notification, and instruct the EMS not to hesitate to alert the stroke team, even if there is still the possibility of other diagnoses. Second, some neurological signs were easily overlooked by the EMS at the scene, and so we have to provide feedback and continually educate the EMS with regard to frequently-missed signs of LVO, such as conjugate deviation, aphasia, and unilateral spatial neglect. Additionally, considering the high performance of recent prehospital scales for LVO including conjugate deviation, we should highlight the importance of EMS staff recognizing the presence of eye deviation correctly in the prehospital setting. Third, some posterior circulation LVO patients may develop severely disturbed consciousness or convulsive seizure as their first symptom, so these could be an atypical clinical presentation of LVO^21–23^. It would be inefficient to bring all patients with these uncommon clinical signs to stroke centers, as this may lead to overload for stroke centers as well as delaying an appropriate response to time-sensitive diseases other than stroke in the emergency department. Further studies will be required to identify the clinical features that distinguish stroke from other etiologies in patients with convulsive status or disturbance of consciousness.

This study had several limitations. First, we should be careful about assessing the comparability between the Hotline group and the Non-hotline group. Intrinsically, it is assumed that there were some clinical features which made it difficult for the EMS to suspect stroke correctly. However, patient characteristics, such as mRS before onset and NIHSS on admission, were similar between the two groups. Further, some comorbidities related to unfavorable patient outcomes were even more common in the Hotline group. Second, there was a potential selection bias as we were unable to evaluate LVO patients who did not undergo EVT. It is not possible to describe the effect of prenotification in all LVO cases including these patients. Third, it may be difficult to generalize our results to other communities, as the protocol was designed to fit EMS systems in Kobe City. When implementing the results in different communities, it is essential to consider differences in individual circumstances such as stroke management systems and geospatial location. Fourth, we could not completely understand the mechanism underlying the fact that good neurological outcomes were higher in the Hotline group than in the Non-hotline group, as the actual difference in time metrics would not be enough to explain this. Further, although the site of occlusion was not statistically significant in the multivariate analysis, it is clinically reasonable to consider that occluded vessels such as VA-BA occlusions must be related to the results of our study. Ideally, further studies with an adequate sample size are required to assess the relationship between prehospital stroke notification and patient outcomes stratified by site of occlusions. Finally, improved outcomes might have been related to unmeasurable factors other than prehospital notification. In particular, indication bias may be present. The EMS might tend to use the hotline in an uncomplicated case more frequently, but hesitate to use it in complicated ones. If there were such a bias, it would make patient outcomes in the Hotline group appear to be better than they actually were. Further, more patients in the Hotline group received tPA than the Non-hotline group. It is beyond the scope of our study to speculate the mechanism underlying the relationship between prehospital stroke notification and frequency of tPA administration. However, one possible explanation is that patients in the Hotline group had shorter onset to hospital time and therefore extended the thrombolysis window.

In conclusion, prehospital notification of stroke by the EMS may contribute to improved clinical outcomes in LVO patients who received EVT. To reduce the number of patients who are transported without suspicion of stroke and establish a more efficient prehospital stroke management system, it is important to understand the clinical features of LVO patients, and to provide the EMS with appropriate on-going feedback and education.

## Data Availability

The datasets analyzed during the current study are not publicly available but are available from the corresponding author on reasonable request.

## Abbreviations

EVT: Endovascular therapy
t-PA: Tissue plasminogen activator
LVO: Large vessel occlusion
EMS: Emergency medical services
mRS: Modified Rankin Scale
NIHSS: National Institute of Health Stroke Scale
ASPECTS: Alberta stroke program early computed tomography score
TICI: Thrombolysis in cerebral infarction
